# SiMa Cells for a Serotype Specific and Sensitive Cell-Based Neutralization Test for Botulinum Toxin A and E

**DOI:** 10.3390/toxins9070230

**Published:** 2017-07-20

**Authors:** Nicola Bak, Shalini Rajagopal, Paul Stickings, Dorothea Sesardic

**Affiliations:** Division of Bacteriology, National Institute for Biological Standards and Control (NIBSC), A Centre of the Medicines and Healthcare Products Regulatory Agency, Hertfordshire EN6 3QG, UK; nicola.a.bak@gmail.com (N.B.); shalini.rajagopal@nibsc.org (S.R.); paul.stickings@nibsc.org (P.S.)

**Keywords:** SiMa cells, BoNT/A, BoNT/E, cell-based assay, toxin neutralization test (TNT), SNAP-25, capture ELISA, immunodetection

## Abstract

Botulinum toxins (BoNTs), of which there are seven serotypes, are among the most potent neurotoxins, with serotypes A, B and E causing human botulism. Antitoxins form the first line of treatment for botulism, and functional, highly sensitive in vitro methods for toxin neutralization are needed to replace the current in vivo methods used for determination of antitoxin potency. In this preliminary proof of concept study, we report the development of a neutralization test using the neuroblastoma SiMa cell line. The assay is serotype specific for either BoNT/A or BoNT/E, which both cleave unique sequences on SNAP-25 within SiMa cells. The end point is simple immunodetection of cleaved SNAP-25 from cell lysates with antibodies detecting only the newly exposed sequence on SNAP-25. Neutralizing antibodies prevent the toxin-induced cleavage of SNAP-25. The toxin neutralization assay, with an EC50 of ~2 mIU/mL determined with a standardized reference antiserum, is more sensitive than the mouse bioassays. Relevance was demonstrated with commercial and experimental antitoxins targeting different functional domains, and of known in vivo neutralizing activities. This is the first report describing a simple, specific, in vitro cell-based assay for the detection of neutralizing antibodies against BoNT/A and BoNT/E with a sensitivity exceeding that of the mouse bioassay.

## 1. Introduction

Botulism is a rare but life-threatening disease caused by neurotoxins produced by several strains of genus Clostridium (*Clostridium botulinum*, *C. butyrricum*, *C. barati* and *C. argentinensis*) [1,2]. Of the seven known serotypes (and more than 40 subtypes) identified to date, serotypes, A, B, E and, rarely, F, are associated with causing human botulism, with several cases of infant, food or drug-user associated botulism reported every year. Botulinum toxins (BoNT)/A and BoNT/E are considered the most lethal biological substances with a human lethal dose estimated at approximately 1 ng/Kg and 1 µg/Kg by parenteral or oral routes, respectively [3].

All currently known serotypes of botulinum toxin are composed of a heavy chain (HC, 100 kDa) and a light chain (LC, 50 kDa), linked together by a disulphide bond, both of which are essential for biological activity [4,5,6,7]. The C-terminal domain of the BoNT/A and BoNT/E HC binds first to a ganglioside on the cell surface and then to a different segment of the luminal loop of a protein receptor on synaptic vesicle glycoprotein (SV2) [4]. The toxin-dual receptor complex then enters the neuron via endocytosis. The acidification triggers a conformational change leading to formation of a channel in the endocytic vesicle membrane to facilitate translocation of the LC into the cytosol and reduction of the disulfide bond linking the HC and LC [5,6]. The released and refolded LC is a catalytically active zinc endopeptidase that cleaves and inactivates Soluble N-ethylmaleimide Sensitive Fusion Attachment Protein Receptors (SNARE), which are essential for neurotransmitter release [7]. This endopeptidase activity of botulinum toxins is highly serotype specific, with each toxin targeting a unique position within three key SNARE proteins associated with fusion of the synaptic vesicle with synaptic membrane. BoNT/A and BoNT/E both cleave SNAP-25 1–206 (synaptosomal associated protein of molecular mass 25 kDa), at positions Q197–R198 and R180–I181, respectively [2,7]. Subsequently, the extracellular release of neurotransmitter into the neuromuscular junction is blocked resulting in a flaccid muscular paralysis that can eventually lead to death.

Effective treatment for botulism involves the timely administration of neutralizing antitoxin and several commercial preparations are available for treatment of humans [8], including infant botulism [9]. New countermeasures in development include human or humanized recombinant monoclonal antibodies [10,11,12,13]. The traditional mouse lethality or systemic toxicity neutralization test for determination of the potency of therapeutic antitoxins has been considered at the forefront for replacement on ethical grounds [14]. Refinements to the lethal assay, such as local flaccid paralysis or digit abduction score in mice, offer enhanced sensitivity and are more humane alternatives [15,16] since mice do not experience systemic toxicity. Another approach - the mouse phrenic nerve-hemidiaphragm assay—is an ex vivo method performed on tissues from mice that does not involve the use of live animals [17]. This relatively rapid approach currently provides the most sensitive assay available for determination of functional botulinum toxin antibodies, but suffers from use of complex equipment, technically challenging dissection, low throughput, and continued reliance on animals for harvest of tissue.

Biochemical assays detecting LC endopeptidase activity have been developed utilizing antibodies to a cryptic sequence on SNAP-25 that is exposed by the action of BoNT/A or BoNT/E. This approach allowed for very sensitive and serotype-specific detection of toxin [18] and could be modified for detection of antibodies inhibiting toxin endopeptidase activity [19,20]. However, potency estimates expressed in IU/mL relative to the reference antitoxin were always lower in the biochemical assay compared to potencies determined in vivo [19], and it was assumed that only some of the neutralizing antibodies were detected, specifically those interacting with the toxin enzyme activity domain. However, biochemical assays proved useful for selection of single chain variable antibody fragments (scFv) targeting BoNT LCs prior to their conversion to full IgG. The in vitro assay was used at this stage because the scFv antibody fragments could not be characterized in vivo due to their short half-life [21]. An antibody directed against BoNT/E LC, which was selected using the biochemical assay (based on in vitro inhibition of the endopeptidase activity), proved to have a highly neutralizing potency in vivo [13]. Similarly, antibodies against BoNT/A and BoNT/B LCs, which were also selected by in vitro assays [22], were neutralizing in vivo when presented in combination with antibodies against the HC of the corresponding toxin serotype [12].

More recently, Rosen et al. [23] reported a novel in vitro potency test for anti-botulinum B antitoxins, incorporating enzyme activity and a receptor binding step in which they were able to obtain comparable potencies to those determined by in vivo bioassay. The concept of this biochemical assay is to mimic two fundamental steps in botulinum intoxication interaction: receptor binding and catalytic activity. Such an assay cannot reflect all the events required for toxin activity in vivo, particularly the complex dual-receptor interaction, internalization step and persistence of toxin within the cell, which can all contribute to toxin potency [24]. Furthermore, different BoNTs interact with distinct protein receptors and are dependent on non-protein components (the polysialoganglioside) for high affinity internalization [7]. This complex mechanism is considered difficult to fully reconstruct in biochemical methods, thus limiting their applicability to detect toxin neutralizing antibodies.

Neuronal cell-based assays offer distinct advantages over existing animal models, not only for detection of toxin, but also for selecting potentially neutralizing anti-botulinum toxin antibodies. These in vitro models incorporate all key steps in toxin activity: binding, internalization, low pH driven translocation and cleavage of specific substrate associated with neuronal transmission. The human neuroblastoma SiMa cell line has been described as having a high degree of differentiation and high capacity for synthesis of neurotransmitters [25]. Such cells have already been successfully used for development of a cell-based potency assay for a commercial product of BoNT/A toxin, BOTOX^®^, as reported by Allergan [26]. The advantage of SiMa cells is their relatively high sensitivity to BoNT/A, even when not differentiated into a fully neuronal phenotype, which considerably reduces the duration of the assay. In addition, they are known to express a high level of SNAP-25, the intracellular target protein for BoNT/A and E toxins, and were confirmed to increase sensitivity to BoNT/A in the presence of GT1b [26,27]. High sensitivity is not shared by many other wild type neuronal cell lines which generally suffer from lower sensitivity despite long differentiation protocols [24] in comparison to primary neurons [28] and neurons derived from stem cells [29]. Moreover, use of primary cells or differentiated stem cells is considered less desirable for routine use within a quality control laboratory setting. In this preliminary, proof of concept study we have investigated the use of commercially sourced SiMa cells, differentiated with a rapid protocol, combined with antibody reagents detecting only BoNT/A- or BoNT/E-cleaved SNAP-25 [18,30], for sensitive and simple detection of toxin neutralizing antibodies.

## 2. Results

### 2.1. Treatment of SiMa Cells and Sensitivity to BoNT/A and BoNT/E

**Figure 1:**
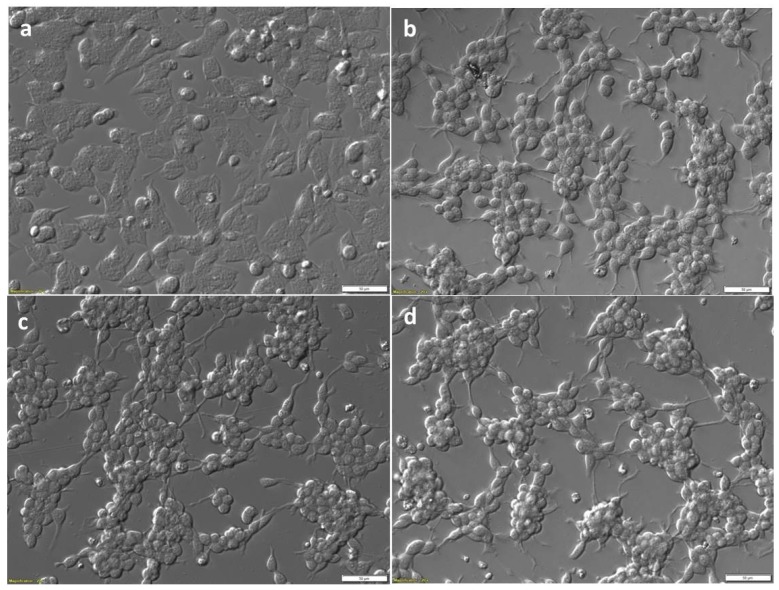
Bright field relief contrast images of SiMa cells pre- and post-differentiation, and post-treatment with BoNT. SiMa cells were initially cultured in basal medium (**a**), and then differentiated in serum-free medium on Biocoat™ 6-well plates in the presence of 25 µg/mL GT1b for 72 h (**b**). Differentiated cells after treatment with either BoNT/A (**c**) or BoNT/E (**d**), both at 200 LD50/mL for a further 48 h. Scale bars represent 50 µm.

Morphology observed by light microscopy of SiMa cells before (Figure 1a) and after three days differentiation (Figure 1b) with the protocol described in Section 4.3. This fast differentiation protocol led to cells which were strongly attached to the wells of the tissue culture plates with high density. There were clearly visible changes in cell morphology after differentiation, with elongated connections and changes towards motor neurons or neuron-like cells in some, but not all the cells. This morphology is similar, although less extensive, when the neuronal cells are differentiated in the presence of retinoic acid (RA) [31]. The fast 3 day differentiation protocol, previously reported with adequate sensitivity for BoNT/A [26,27], proved optimum for generation of high attachment and density of cells required for immunodetection of cleaved SNAP-25 from the cell lysates. Morphology of SiMa cells after 48 h treatment with 200 LD50/mL (87 pg/mL; 6 pM) BoNT/A (Figure 1c) and 200 LD50/mL (100 pg/mL; 8 pM) BoNT/E (Figure 1d) was not different to that seen for differentiated SiMa cells prior to toxin treatment.

**Figure 2:**
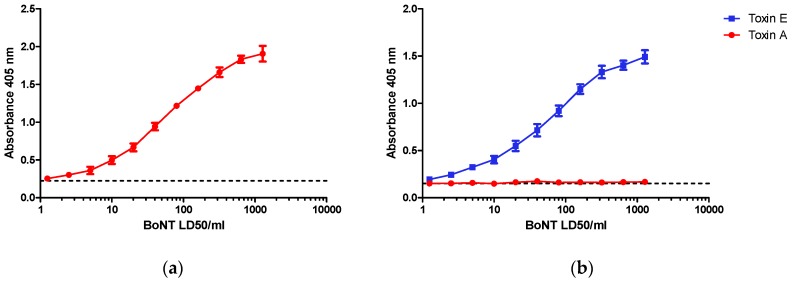

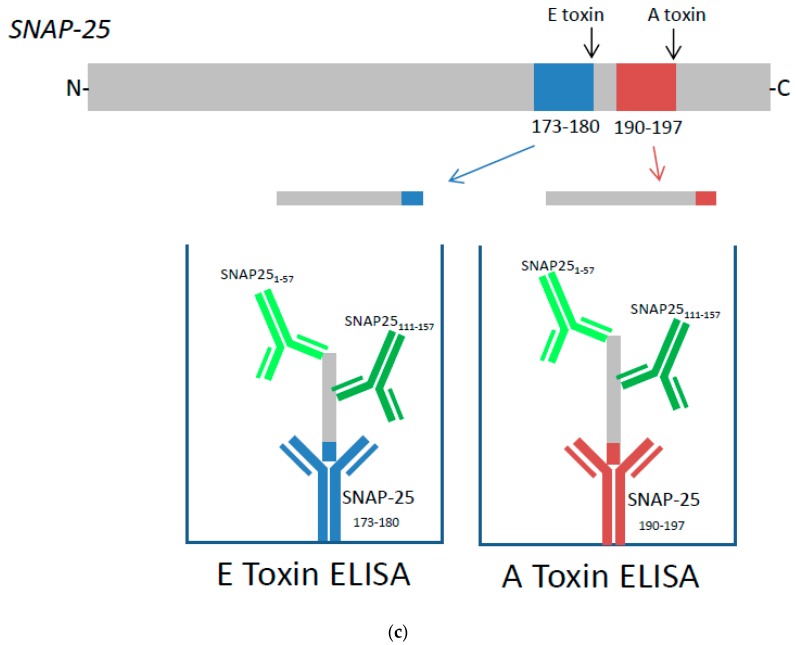
Dose dependent detection of cleaved SNAP-25 specific for (**a**) Botulinum toxins (BoNT)/A and (**b**) BoNT/E toxins. SiMa cells were differentiated for 3 days on 96-well tissue culture plates and treated with either purified BoNT/A or BoNT/E toxins in a range of concentrations between 1–1280 LD50/mL (~5 pg/mL to ~5 ng/mL). After 48 h exposure, cells were lysed and subjected to toxin specific capture ELISA for detection of either BoNT/A (**a**) or BoNT/E (**b**) cleaved SNAP-25. Dotted line indicates controls where cells were not exposed to toxins. Results are from one typical assay performed on at least three independent occasions and each data set is a mean from four individual wells ±SD. (**c**) Schematic overview of capture ELISA for BoNT/A and BoNT/E: BoNT/A cleaves SNAP-25 between amino acids 197 and 198 and the cleavage product is captured using a specific neo-epitope antibody raised against a peptide corresponding to amino acids 190–197 of SNAP-25 (SNAP-25_190–197_). BoNT/E cleaves SNAP-25 between amino acids 180 and 181 and the cleavage product is captured using a specific neo-epitope antibody raised against a peptide corresponding to amino acids 173–180 of SNAP-25 (SNAP-25_173–180_). The captured cleavage product is then detected using two polyclonal detection antibodies that bind to two distinct sites, SNAP-25_1–57_ and SNAP-25_111–157_.

SiMa cells after three days of differentiation were incubated for 48 h with purified BoNT/A or BoNT/E at a range of concentrations from 1–1280 LD50/mL (0.2–256 LD50/dose). Cleaved SNAP-25 was detected by capture ELISA using antibodies selective for BoNT/A (Figure 2a) or BoNT/E (Figure 2b) detection. For both toxins, there was a clear dose response in specific SNAP-25 cleavage in the range of concentrations selected for the study with LOD close to 10 LD50/mL, representing 2 LD50/dose or 8 to 10 pg of toxin per dose. A significant response (*p* < 0.05) was detectable with doses of 10 LD50 and higher when compared to control wells containing cells alone in the absence of BoNT/A. The respective dose response curve for BoNT/A and BoNT/E were very similar, with an EC50 of approximately 100 LD50/mL (20 LD50/dose) for both serotypes and a maximum response at a dose of ~1000 LD50/mL. Dose response curves were consistent between experiments although the maximum absorbance at 405 nm did vary with different batches of cells. Positive and highly reproducible detection of SNAP-25 cleavage was evident only in the presence of the respective toxins. BoNT/A at comparable concentrations had no effect (i.e. no visible increase in detection signal) in the BoNT/E assay (Figure 2b) despite the fact that this toxin also cleaves SNAP-25, but at a different site. Specificity of the antibodies used in the assay and assay design is shown in Figure 2c.

### 2.2. BoNT/A Neutralization Assay

**Figure 3:**
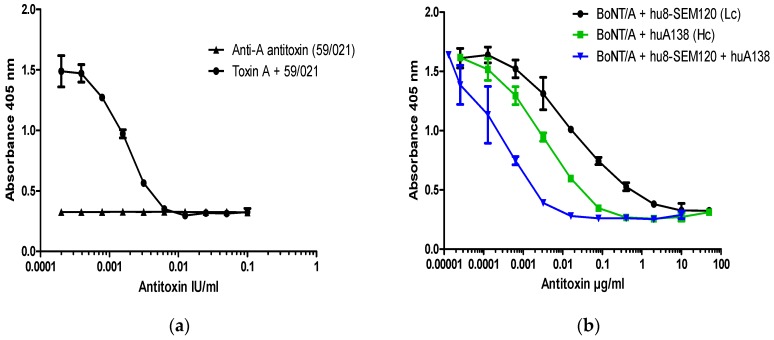
Dose dependent inhibition of BoNT/A cleavage of SNAP-25 from SiMa cells by (**a**) reference polyclonal and (**b**) humanized recombinant monoclonal antibodies against BoNT/A. SiMa cells were differentiated for 3 days on 96-well tissue culture plates and treated with a mixture of purified BoNT/A toxin (200 LD50/mL or 40LD50 per well) and (**a**) reference antitoxin for BoNT/A (NIBSC product code 59/021) in the range of concentrations between 0.1 IU and 0.1 mIU or (**b**) humanized monoclonal antibodies targeting heavy chain (HC) or the light chain (LC) of BoNT/A [12]. After 48 h exposure to the corresponding mixtures, cells were lysed and subjected to capture ELISA for detection of BoNT/A cleaved SNAP-25. Results are from one representative assay where each data set is the mean from two individually treated wells ±SD. Reference antitoxin for BoNT/A (NIBSC product code 59/021) was also included as a negative control in the absence of BoNT/A (**a**).

All BoNT/A neutralization assays were performed with 200 LD50/mL of pure BoNT/A which corresponds to 40 LD50/dose (6 pM or ~150 pg of pure BoNT/A/dose). This concentration of toxin was fully neutralized with 10 mIU/mL (2.0 mIU/dose) of type A reference antitoxin (National Institute for Biological Standards and Control (NIBSC) product code 59/021) (Figure 3a). Neutralization of a fixed dose of BoNT/A by antitoxin A resulted in a significant dose response over the range between 10 mIU/mL and 0.5 mIU/mL, with an EC50 at ~2 mIU/mL (0.4 mIU/dose) and lowest level of detection at ~0.5–0.1 mIU/mL (Figure 3a).

To explore the applicability of the SiMa cell neutralization assay, well characterized monoclonal antibodies directed against the HC (hu8A1HC38) and the LC (hu8SEM120-IIIC1) of BoNT/A were included in the test. These antibodies were previously characterized for their neutralization properties in two mouse bioassays in vivo [12]. Both antibodies against BoNT/A were able to dose-dependently prevent specific SNAP-25 cleavage induced by 200 LD50/mL (40 LD50/dose) of BoNT/A. The antibody against LC (hu8SEM120-IIIC1) completely neutralized BoNT/A activity at 10 µg/mL (2.0 µg/dose), whereas antibody against HC (huA1HC38) was at least 10 times more potent and fully neutralized the BoNT/A at 1 µg/mL (0.2 µg/dose). The minimum level of detection for antibody against the LC and the HC was ~1 pg/mL and ~0.1 pg/mL, respectively. A mixture of equal concentrations of the antibodies against the HC and LC completely neutralized BoNT/A activity at a dose of 0.01 µg/mL (2.0 pg/dose) (Figure 3b), with the minimum level of detection at ~0.02 pg/mL. Antibody combination is therefore approximately 100- and 1000-times more potent than the individual antibodies against the HC and the LC, respectively.

**Figure 4:**
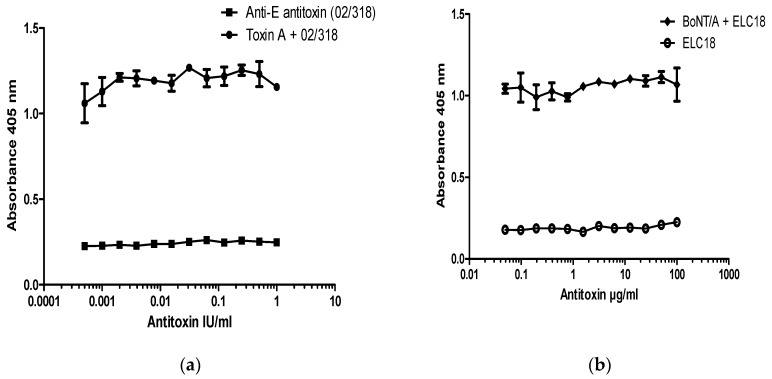
Specificity of SiMa cell toxin neutralization assay for BoNT/A. SiMa cells were differentiated for 3 days on 96-well tissue culture plates and treated with a mixture of purified BoNT/A toxin (200 LD50/mL or 40 LD50 per well) and (**a**) a reference antitoxin for BoNT/E (NIBSC product code 02/318) in the range between 1 IU and 0.1 mIU or (**b**) humanized recombinant monoclonal antibody targeting the LC of BoNT/E (ELC18) [13]. After 48 h exposure to the corresponding mixtures, cells were lysed and subjected to capture ELISA for detection of BoNT/A cleaved SNAP-25. Results are from a representative experiment where each data set is the mean from two individually treated wells ±SD. Reference antitoxin for BoNT/E (NIBSC product code 02/318) and humanized antibody ELC18 were also incubated with SiMa cells in the absence of BoNT/A as negative controls and were from a single cell reading per dilution.

In order to establish if the neutralization assay is specific for detection of antibodies to BoNT/A, experiments were performed on SiMa cells treated with BoNT/A that had been pre-incubated with BoNT/E antitoxin (Figure 4). There was no neutralization of BoNT/A-induced cleavage of SNAP-25 in the presence of an equine polyclonal antibody to BoNT/E (NIBSC product code 02/318) (Figure 4a) or with a neutralizing monoclonal antibody against the LC of BoNT/E (Figure 4b). These results confirm that neutralization assay for BoNT/A antibodies is serotype specific. Furthermore, when any of the two antibodies were included in the assay alone, in the absence of BoNT/A, they had no effect on SNAP-25 cleavage, and OD values were comparable to the background values observed in the absence of toxin (cell controls) (Figure 4a,b).

### 2.3. BoNT/E Neutralization Assay

**Figure 5:**
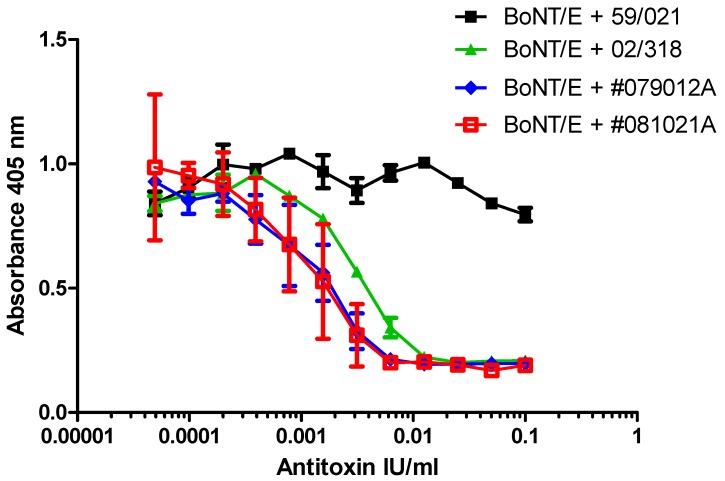
Dose dependent inhibition of BoNT/E cleavage of SNAP-25 from SiMa cells by polyclonal type E antitoxins. SiMa cells were differentiated for 3 days on 96-well tissue culture plates and treated with a mixture of purified BoNT/E toxin (200 LD50/mL or 40 LD50 per well) and reference antitoxin for BoNT/E (NIBSC product code 02/318), or two separate batches of polyclonal trivalent antitoxin (#079012A and #081021A, with assumed potency of >50 IU/mL for antitoxin type E) diluted in the range between 0.1 IU/mL and 0.05 mIU/mL. Reference antitoxin for BoNT/A (NIBSC product code 59/021) was included as a negative control. After 48 h exposure to the corresponding mixtures, cells were lysed and subjected to capture ELISA for detection of BoNT/E cleaved SNAP-25. Results are from a single experiment where each data set is the mean of two individually treated wells ±SDs. SiMa cells were also treated with the antitoxins in the absence of BoNT/E and no signal was observed in the capture ELISA (Supplementary Materials Figure S1).

BoNT/E neutralization assays were performed with 200 LD50/mL of BoNT/E, which corresponds to 40 LD50/dose (~8 pM or 200 pg/dose of pure BoNT/E). This concentration of toxin was fully neutralized with 10 mIU/mL (2 mIU/dose) of type E reference antitoxin (NIBSC product code 02/318). Pre-incubation of BoNT/E with type E reference antitoxin resulted in a significant dose-dependent reduction in detection of specific SNAP-25 cleavage over the range between 0.5 mIU/mL to 10 mIU/mL, with an EC50 of approximately 2 mIU/mL (0.4 mIU/dose). The BoNT/A antitoxin (NIBSC product code 59/021), even at a 10-fold excess of 100 mIU/mL, did not prevent cleavage of SNAP-25 by BoNT/E to any notable extent (Figure 5).

Dose dependent inhibition of BoNT/E-specific cleavage of SNAP-25 within SiMa cells was also confirmed with two different batches of commercially sourced trivalent polyclonal sera with defined neutralizing activity for BoNT/E of >50 IU/mL (Figure 5). The dose response for the trivalent antitoxin preparations was parallel to the reference antitoxin allowing statistically valid estimates of relative potency to be calculated. Relative to the reference standard for type E antitoxin (NIBSC product code 02/318), and using parallel line analysis, the potency estimate and 95% confidence intervals was calculated as 119 (95–159) IU/mL for batch #079012A and 125 (82–190) IU/mL for batch #081021A. The results confirmed the expected specification of >50 IU/mL. Furthermore, these estimated potencies were within the range determined by the mouse paralysis assay [15], where potency of between 60 and 170 IU/mL was calculated relative to the same reference standard (NIBSC product code 02/318).

When any of the four equine antibody preparations (reference type A antiserum, 59/021, reference type E antiserum, 02/318, or two different batches of trivalent antitoxin) were incubated with SiMa cells in the absence of BoNT/E toxin, there was no interference in the assay and OD values were comparable to the control cells with no BoNT/E toxin (Supplementary Materials Figure S1).

## 3. Discussion

In this proof of concept study, we describe an in vitro cell-based assay for the detection of antibodies against botulinum neurotoxin serotypes A and E. The assay end point is the simple immunodetection of truncated SNAP-25 peptides from lysates of SiMa cells previously incubated with mixtures of botulinum neurotoxin and antibody. Specificity of the assay described in this report is dependent on the capture antibodies which were designed to detect only the BoNT/A- or BoNT/E-cleaved SNAP-25. We have previously used the same antibodies for development of an endopeptidase activity ELISA assay and functional dual-coated ELISA assay using synthetic or recombinant SNAP-25 [18,30]. In this study, we confirmed the results of Fernadez-Salas et al., [26] on the suitability of SiMa cells for detection of BoNT/A activity and report that this approach is also suitable for specific detection of BoNT/E activity. The dose response curves for the two toxins are similar, showing dynamic increase over the range of between ~10 LD50/mL and 500 LD50/mL, and with an EC50 at approximately 100 LD50/mL. Dose response curves showed significant regression in all assays and were comparable between experiments, although the maximum response (OD 405 nm) did vary for different batches of cells. However, it should be noted that in routine use, the potency of a toxin or antitoxin would be expressed relative to a reference preparation which would control for differences in the absolute OD values obtained for any single experiment. For both toxins, a concentration of 200 LD50/mL (6–8 pM; 40 LD50/dose) was selected for neutralization studies which corresponded to the upper end of the linear part of the dose response curve.

Cell-based assays for detection of BoNTs are considered the only in vitro strategy suitable to replace the mouse bioassay as recommended by experts in the field [32]. It was considered relevant that any method for complete replacement of animals will be required to reflect all events leading to intoxication in vivo. In her excellent review, Pellett [24] describes the current status of cell-based assays for detection of BoNTs. The general consensus is that primary neuronal cells and stem cell-derived neurons offer higher sensitivity compared to any continuous cell lines irrespective of the end point or differentiation protocol used [24]. The notable exception is the human neuroblastoma SiMa cell line, which, even after a short 3-day differentiation, offers sensitivity comparable to that of the mouse bioassay for potency evaluation of BoNT/A in BOTOX^®^ pharmaceutical products [26]. BoNTs are known to enter neuronal cells by dual receptor binding involving gangliosides and protein, and cell lines are considered to be less sensitive to BoNTs, even when they express the relevant protein receptor, because they have a low level of gangliosides compared to primary cells. Addition of ganglioside, particularly GT1b, was found to increase sensitivity to BoNT/A (more effectively than GD1a or GM1) when added to Neuro 2A or to human adrenergic SK-N-SH neuroblastoma cells [33], SH-SY5Y cells [31] and SiMa cells [27]. Whereas future studies could explore if different mixtures of polysialogangliosides can improve the sensitivity of SiMa cells, the focus of this study was to establish the feasibility of the SiMa cell model as a BoNT neutralization test. We focused on the use of ELISA as an end point because it is applicable to high-throughput formats and is a more quantitative method compared to Western Blotting [34].

A potential limitation of the cell-based toxin neutralization test is the possibility of toxin-induced cytotoxicity. Whereas BoNTs are neither cytotoxic or causative agents of axonal degeneration in vivo, toxicity in vitro has been reported for BoNT/C on various neuronal cell lines and rat primary neuronal cells [35]. Studies by Peng et al. [36] confirmed that BoNT/E at >300 pM and two weeks exposure with rat hippocampal neurons can induce axonal degeneration. Concentrations below 100 pM were non-toxic. In our studies, we show that neither BoNT/A or BoNT/E induced morphological changes to differentiated SiMa cells at concentrations of ~6–8 pM, suggesting that these toxins are not cytotoxic in the conditions used in the cell-based assay.

Cell-based neutralization assays have been developed and used previously for specific detection of neutralizing serum antibodies against several other bacterial toxins, e.g. anthrax [37] and toxins from *Clostridium difficile* [38] and *Clostridium perfringens* [39]. Use of cell-based assays for detection of low levels of botulinum toxin neutralizing antibodies, such as in patients undergoing botulinum toxin therapy, is limited. For such an approach, particularly sensitive toxin neutralization methods are required due to the very low levels of circulating antibodies—approximately 1 mIU/mL—that can cause resistance to further toxin therapy [19]. Embryonic spinal cord neurons from rats offer high sensitivity and have been applied to the detection of botulinum antibodies in human serum [40] with a standardized reference serum to assess sensitivity in IU/mL [41]. However, primary cells still rely on the use of animals and require substantial and relatively lengthy manipulation making them less attractive for routine use [24]. In these previously reported neutralization assays, detection of intracellular substrate (SNAP-25 or VAMP2) was by Western Blotting or by assessment of stimulated (^3^H) glycine release. The ELISA end point method described here offers significantly higher throughput capacity yet provides equivalent sensitivity to that previously reported with embryonic spinal cord cells for standardized reference antiserum [41]. The neutralization assay described here can therefore provide an alternative to existing in vivo or ex vivo methods [15,17] when antibody detection of high sensitivity is required. No other simple in vitro method is sensitive enough for detection of antibody-induced resistance in patients during long term toxin therapy [42].

The cell-based neutralization assay can also be used for screening and characterization of the new generation of antibody countermeasures. Short chain fragment antibodies (scFvs or scFv-Fc) targeting different functional domains of the toxin can be screened in the same assay without the use of animals. Here we confirmed the same ranking order of neutralizing potency for two recombinant monoclonal antibodies determined in the cell-based neutralization test and in the previously reported mouse protection models in vivo [12]. In our previously published studies, antibody to the LC of BoNT/A was only able to delay or partially protect animals from toxin lethality or paralysis, but the combination of two antibodies (one directed against the LC and one against the HC) was at least 100 times more effective, which was also confirmed by toxin neutralization on SiMa cells. Utilizing such an entirely in vitro approach during production of the next generation of recombinant antibodies would considerably reduce the cost and limit the use of animals. Animals may only need to be used during pre-clinical studies where information other than functional neutralization needs to be established. Information on antibody clearance rate and half-life cannot (currently) be addressed by in vitro methods [43].

Previous studies using cells to confirm toxin neutralization properties have not compared the results with in vivo neutralization data, but rather were often performed to confirm positive neutralization [10,11,40]. Studies by Rosen et al. [23] confirmed that antitoxin potency determined in vitro for botulinum type B toxin correlated highly with that determined by the standard in vivo mouse assay for a range of polyclonal antitoxin preparations. However, the preparations tested were all of high potency, in the range between 1000–4500 IU/mL, and the assay is only applicable to detection of antibodies against BoNT/B toxin. Furthermore, the method described by Rosen et al. [23] involves detection of only two fundamental steps in botulinum toxin action - receptor binding and enzyme activity - and the complexity of receptor interaction and the toxin entry step is not reflected. We have previously reported a similar approach for sensitive detection of BoNT/A toxin [30], but have not applied this approach for detection of neutralizing antibodies.

The approach described in this study could be easily expanded to other clostridial neurotoxins provided that SiMa cells can be stably transfected with vesicle-associated membrane protein (VAMP2), an intracellular substrate for several other serotypes of botulinum neurotoxin and tetanus toxin, which is not naturally expressed in this cell line [44].

In summary, SiMa cells have been used to develop serotype specific toxin neutralization assays for BoNT/A and BoNT/E, with sensitivity better than that of the conventional mouse neutralization test. The assays have a minimum level of detection of between 0.05 and 0.1 mIU/mL, (EC ~2 mIU/mL) which is 100-times and 10-times more sensitive than the mouse lethal and paralysis neutralization tests, respectively. No other simple, specific in vitro cell-based assay for the detection of neutralizing antibodies against BoNT/A and BoNT/E, with a low detection level, has been reported to date.

## 4. Materials and Methods

### 4.1. Toxins

Purified haemagglutinin-free BoNT/A1 and BoNT/E1 were purchased from Metabiologics (Madison, WI, USA) and are fully described in previous publications [12,13,17]. Both preparations were diluted to 20,000 LD50/mL (87 ng/mL for BoNT/A and 100 ng/mL for BoNT/E) in Gelatine (0.2% *w*/*v*) Phosphate (50 mM di-sodium hydrogen orthophosphate) Buffer pH 6.5 (GPB). Toxins were stored in frozen aliquots at −80 °C prior to use.

### 4.2. Antibodies

Botulinum antitoxin reference standards: serotype A, equine (NIBSC product code 59/021) with activity of 2000 IU/ampoule, and serotype E, equine (NIBSC product code 02/318) with activity of 197 IU/ampoule.

Test antitoxins: Humanised recombinant IgGs targeting HC (A1HC38) and LC (SEM120-IIIC4) of BoNT/A, or LC (ELC18) of BoNT/E, were produced as part of the EU Framework 7 AntiBotABE project [45] and fully characterized as reported elsewhere [12,13]. Commercial polyvalent F(ab)^2^ fraction from horse serum was supplied by Novartis (Marburg, Germany) (lots #081021A and #079012A).

Capture ELISA antibody reagents: BoNT/A and BoNT/E cleavage site-specific anti-peptide antibodies against SNAP-25_190–197_ and SNAP-25_173–180_ were reported and characterized previously [18,46]. Affinity purified polyclonal antibody against these same sites on SNAP-25 were custom made by Biotrend (Köln, Germany) and were used in this study. For detection, in-house anti SNAP-25 antibodies against SNAP-25_1–57_ and SNAP-25_111–157_ were raised in sheep as previously reported [30] and were used for signal amplification in the capture ELISA assay (see Figure 2c).

### 4.3. Preparation of SiMa Cells and Neutralization Assays

SiMa cells (human neuroblastoma cell line) were purchased from the German Collection of Microorganisms and Cell Cultures (DSMZ, Braunschweig, Germany, Catalogue #ACC 164) and maintained in growth medium recommended by the supplier on BioCoat™ collagen IV coated T175 flasks (VWR). Growth medium: RPMI-1640 (+l-glutamine), 10% fetal bovine serum (FBS, heat inactivated), 0.1 nM non-essential amino acids, 10 mM HEPES, 1 nM sodium pyruvate, 50 IU/mL penicillin and 50 μg/mL streptomycin. For a rapid and robust differentiation in cell-based assays, SiMa cells were exposed for 5 min to TrypLE Express, washed with growth medium and transferred to 96-well Poly-d-Lysine pre-coated tissue culture plates (Corning, BioCoat™ from VWR, Lutterworth, UK) at 5 × 10^4^ cells per well in 100 µL of differentiation medium containing freshly prepared 25 µg/mL of GT1b (Sigma, Dorset, UK). Differentiation medium: growth medium excluding 10% FBS and including 1× N-2 supplement liquid and 1× B-27 serum-free supplement. All the tissue culture reagents were from Thermo Fisher Scientific (Hemel Hempstead, UK) unless stated otherwise. Cells were incubated in a 5% CO_2_ incubator at 37 °C for three days prior to treatment. The method is based on the previous publication [26].

After three days, the differentiation medium was carefully removed and purified BoNT/A or BoNT/E toxin was added to SiMa cells in a 200 µL volume diluted in differentiation medium. The tissue culture plates were then incubated for 48 h in a 5% CO_2_ incubator at 37 °C. Initial dose finding studies included toxin concentrations between 1 and 1280 LD50/mL, and for all toxin neutralization studies, 200 LD50/mL (40 LD50/dose) was used.

In all toxin neutralization studies, the concentrations of test antibodies that could prevent intracellular cleavage of SNAP-25 induced by the fixed dose of either BoNT/A or BoNT/E were determined. Toxin was pre-incubated for 1 h at room temperature (RT) with an equal volume of either reference antitoxin (ranging between 0.1 IU/mL and 0.1 mIU/mL or 0.05 mIU/mL for anti BoNT/A and anti BoNT/E, respectively) or recombinant humanized antibodies (100 µg/mL to 0.01 ng/mL). All dilutions were performed in differentiation medium. The toxin-antitoxin mixture (200 µL) was then added to three day differentiated SiMa cells and plates were incubated for 48 h in a 5% CO_2_ incubator at 37 °C. All treatments of cells were performed in at least duplicate, unless stated otherwise.

For photography, SiMa cells were grown on 6-well BioCoat Poly-D-Lysine pre-coated tissue culture plates at 1 × 10^6^ cells per well. Images were captured before and after differentiation for 3 days and after 48 h treatment with 200 LD50/mL of BoNT/A and BoNT/E toxins, using an Olympus IX71 microscope and cellSens software (v1.12, Olympus, Southend-on-Sea, UK).

### 4.4. Preparation of Cell Lysate and Capture ELISA

After 48 h treatment with either toxin or toxin-antitoxin mixtures, cells were carefully washed with phosphate buffered saline (PBS) and lysed with 100 µL per well of freshly prepared lysis buffer: M-PER with 150 mM NaCl (Mammalian Protein Extraction Reagent, Fisher Scientific 15270241) containing EDTA-free protease inhibitor solution. After 5 min shaking at RT, lysed cells were removed to Eppendorf tubes and clarified by centrifugation (10 min at 10,000× *g*) before use in the ELISA. Unless used on the same day, cell lysates were stored frozen at −80 °C before use.

For the ELISA, 96-well ELISA plates (Nunc, Maxisorb, Thermo Fisher Scientific, Hemel Hempstead, UK) were coated with 50 µL per well of 5–10 µg/mL of affinity purified capture antibody against SNAP-25_190–197_ (BoNT/A assay) or SNAP-25_173–180_ (BoNT/E assay) in carbonate coating buffer (pH 9.6), overnight at 4 °C. After carefully removing buffer by decanting, the plates were blocked with 250 µL per well of 2.5% (*w*/*v*) skimmed milk powder (Marvel, Premier Foods Group, London, UK) in PBST (phosphate buffered saline with 0.05% *v*/*v* Tween-20) (M-PBST or blocking buffer) for 60–90 min at RT. The plates were washed with PBST using a plate washer. Cell lysate (50 µL) was added to the plates, together with 50 µL of M-PBST, and the plates placed on a shaker at 400 rpm for 10 min followed by resting for 90 min at RT. M-PBST (100 µL per well) was added to blank control wells. Plates were washed three times with PBST and detection of captured truncated SNAP-25 was performed by addition of a mixture of sheep anti-SNAP-25 antibodies directed to SNAP-25 amino acids 1–57 and 111–157 in M-PBST at 100 µL per well (1/1000 dilution from stock). After incubation for 90 min at RT, the plates were washed three times with PBST and the reaction visualized with 100 µL per well of commercially sourced rabbit anti-sheep HRP conjugated antibody (Thermo, #31480) in M-PBST and incubated for 90 min at RT. After washing with PBST, 200 µL per well of substrate solution (50 mM citric acid pH 4.0, 0.05% *w*/*v* ABTS (2,2′-azino-bis(3-ethylbenzothiazoline-6-sulfonic acid) diammonium salt, and 0.05% *v*/*v* of a 30% *w*/*v* hydrogen peroxide solution) was added to all wells and the colour allowed to develop at RT for 30 min. Following colour development, the plates were briefly shaken and absorbance read at 405 nm using a Multiskan plate reader (Labsystems, Helsinki, Finland). 

## Supplementary Materials

The following are available online at www.mdpi.com/2072-6651/9/7/230/s1, Figure S1: Supplementary data for Figure 5. showing the response for cells incubated with botulinum antitoxin alone, in the absence of BoNT.
