# Supplementary material for: SiMa Cells for a Serotype Specific and Sensitive Cell-Based Neutralization Test for Botulinum Toxin A and E
Source: Toxins (Basel). 2017 Jul 20;9(7):230. doi: 10.3390/toxins9070230 (PMC5535177; doi:10.3390/toxins9070230)
Supplement: Supplementary file 1 [file toxins-09-00230-s001.pdf]

Toxins **2017**, *9*, 230; doi:10.3390/toxins9070230

Supplementary Materials: SiMa Cells for Serotype Specific and Sensitive Cell-Based Neutralization Test for Botulinum Toxin A and E

Nicola Bak, Shalini Rajagopal, Paul Stickings and Dorothea Sesardic

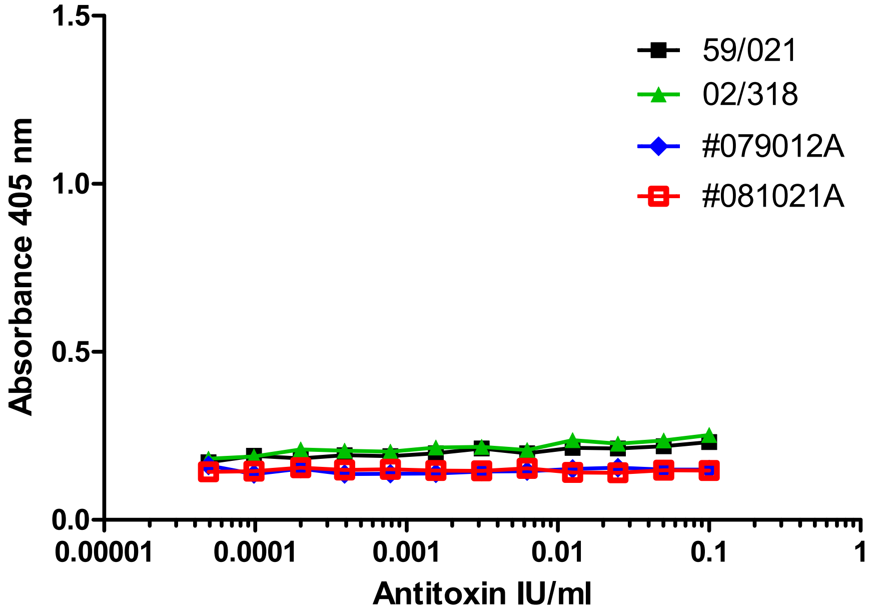

Figure S1. Supplementary data for figure 5 showing the response for cells incubated with botulinum antitoxin alone, in the absence of BoNT. SiMa cells were differentiated for 3 days on 96-well tissue culture plates and treated with botulinum antitoxin diluted in the range 0.1 to 0.00005 IU/ml. Antitoxins used: reference antitoxin for BoNT/E (NIBSC product code 02/318); reference antitoxin for BoNT/A (NIBSC product code 59/021); Two separate batches of polyclonal trivalent antitoxin (#079012A and #081021A). After 48 h, cells were lysed and subjected to capture ELISA for detection of BoNT/E cleaved SNAP-25. Results are from a single titration in a single experiment.
